# Hamartoma of the Finger: A Case Report

**DOI:** 10.5704/MOJ.2507.019

**Published:** 2025-07

**Authors:** SNT Alsagoff, CH Lim, EZF Soh, S Abdullah, J Sapuan

**Affiliations:** Department of Orthopaedics and Traumatology, Universiti Kebangsaan Malaysia, Kuala Lumpur, Malaysia

**Keywords:** hamartoma finger, benign mass finger, hamartoma, hamartoma in hands, benign finger tumour

## Abstract

Hamartomas are benign masses of disorganised tissue native to specific anatomical sites, with a potential for malignant transformation. While they can manifest in various organs, cases involving the hands are exceptionally rare. Hamartomas in the hand have been documented sparingly. To date, there have been no cases of hamartomas in the fingers, hence contributing to the limited body of literature. This case report discusses a hamartoma in the right little finger of an 80-year-old male with a history of a slowly growing painless mass over the course of 5 years. Examination revealed a 1x1 mass with benign features over the proximal interphalangeal joint (PIPJ). A plain radiograph was unremarkable. An excision biopsy was done, and histopathological examination (HPE) revealed a diagnosis of hamartoma. Post-operatively, his wound healed accordingly, and normal function of the finger was achieved.

## Introduction

The term "hamartoma" originates from the Greek word for "error" or "fault" and was first described by Eugen Albrecht in 1904. Albrecht defined hamartomas as tumour-like formations composed of an abnormal mixture of standard components of the anatomical site, differing in amount, structure, and degree of maturity^1^. Hamartomas can appear in various organs and are typically benign, though malignant transformation is possible^2^. Diagnosis is primarily confirmed through histopathological examination (HPE), as clinical examination often reveals similarities with other tumours, such as ganglion cysts or calcified tumours^3^. To date, documented cases of finger hamartomas are rare, with most literature focusing on lipofibromatous hamartomas originating from peripheral nerves, commonly the median nerve^4^.

This report is a case of hamartoma in the right little finger, where clinical examination and radiographs were inconclusive. Intra-operative findings confirmed the mass was not nerve-originated, and HPE confirmed the diagnosis of hamartoma with the presence of predominantly thick and thin-walled blood vessels, mature adipocytes, fibro-myxoid stroma, and scattered nerve bundles.

## Case Report

**Fig. 1:**
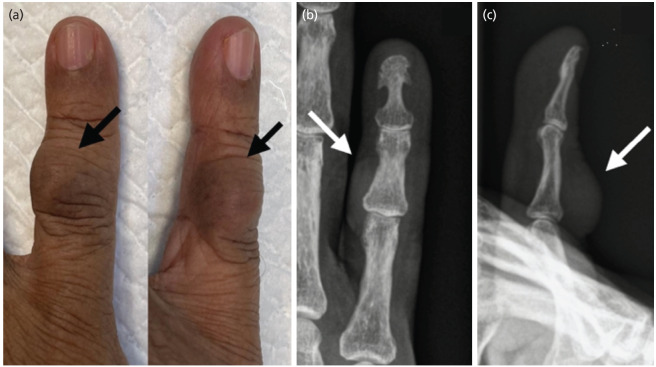
(a) Clinical photograph showing the dorsal aspect of the right little finger with swelling (black arrow) over the PIPJ with no skin changes and smooth surface. (b) Plain radiograph of the right little finger in anterior-posterior view and (c) lateral view showing no bone involvement and soft tissue swelling (white arrow).

An 80-year-old male with a history of hypertension, dyslipidaemia, and chronic kidney disease presented with a painless mass on his right little finger and progressively growing over five years. There was no history of injuries or similar occurrences prior. There were no associated symptoms like reduced joint movement or numbness, and daily activities were unaffected. The mass, measuring 1x1 cm, was located over the proximal interphalangeal joint (PIPJ). There were no skin changes on the round mass. It was soft, non-mobile, non-tender, non-fluctuant, and had a smooth surface (Fig. 1a). Tinel’s sign was negative, and the neurovascular status of the finger was not compromised. A plain radiograph showed no bony erosion (Fig. 1b and 1c). A differential diagnosis of ganglion cyst and epidermoid cyst was made. Hence, no further imaging, such as magnetic resonance imaging (MRI), was done.

**Fig. 2:**
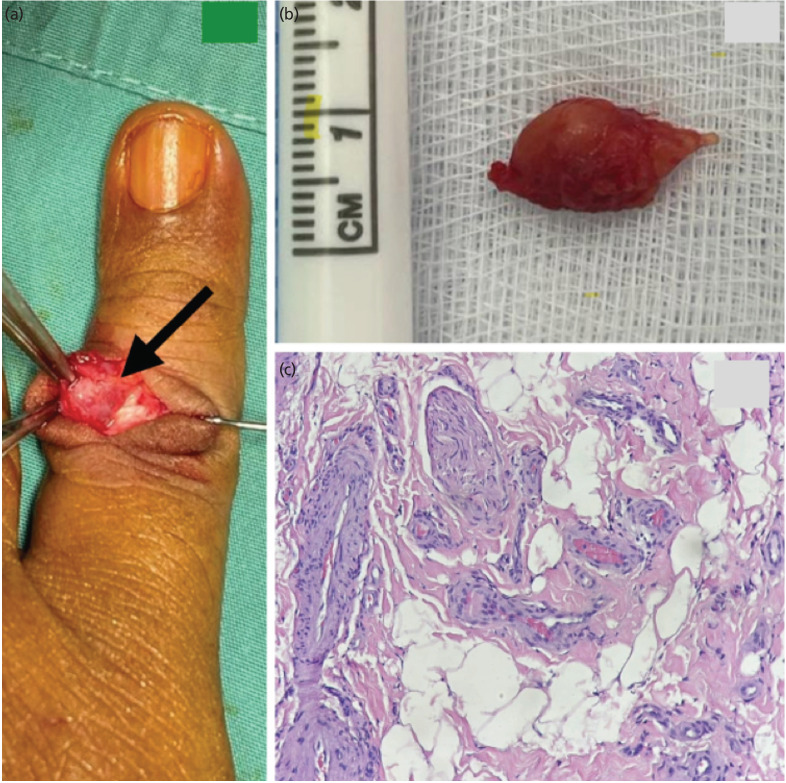
(a) Intra-operative images, showing the mass (black arrow) during excision biopsy, and (b) yellowish-brown, non-mucin-like mass of 0.5x1cm. (c) Histological showed lesion composed of variable-sized blood vessels admixed with mature adipocytes and nerve bundle (H&E stain, x20).

Given the patient's concern, an excision biopsy was performed under local anaesthesia. Informed written consent was obtained from the patient. The mass was then carefully dissected from the surrounding tissues. Intra-operatively, a yellowish-brown, non-mucin-like mass of 0.5x1cm was arising from the extensor tendon sheath, with the extensor tendon and digital nerve appearing healthy (Fig. 2a and 2b). HPE diagnosed hamartoma, revealing predominant thick and thin-walled blood vessels, mature adipocytes, fibro myxoid stroma, and scattered nerve bundles (Fig. 2c).

**Fig. 3:**
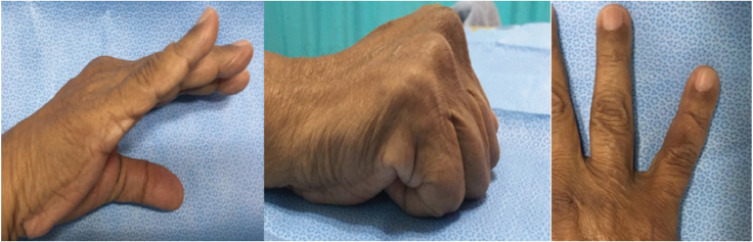
Clinical photograph showing the dorsal aspect of the right little finger at two years follow-up with full range of motion and no recurrence.

Post-operatively, the patient experienced no discomfort or loss of sensation. Two weeks post-operation, the wound healed and the range of motion in the little finger remained full. He was then followed-up for three months, six months, then yearly. The patient was followed-up for two years with no recurrence and full range of motion (Fig. 3).

## Discussion

Hamartomas are benign masses of disorganised tissue native to a specific anatomical site, first described by Eugen Albrecht in 1904. Eugen Albrecht also defined choristomas as malformations of tissues not typically found at that site^1^. Hamartomas can appear in various organs, including the hypothalamus, breast, and lungs^1^. Generally asymptomatic, hamartomas are often discovered incidentally during investigations for other conditions. Complications can arise from infection, infarction, pressure, or obstructions, though malignant transformation is rare^2,5^.

Historically, hamartomas in the hand have been documented sparingly, with a notable case series by Jeffery in 1973, where he presented three cases of hamartoma in the hand^3^. So far, there have been no cases of hamartomas in the fingers unless they originate from peripheral nerves, most commonly the median nerve, ulna nerve, or digital nerve^4^. They are called lipofibromatous hamartoma (LFH), characterised by benign overgrowth of perineural fibro-adipose tissue^4,5^. The case presented here differs as the hamartoma did not arise from the nerves and presented without nerve compression symptoms.

In this case, the patient had a slow-growing, painless mass in the little finger, with no other symptoms. Clinical examination would not give any clue on the diagnosis of hamartoma, as they often resemble other, more common masses such as ganglion cysts or lipomas^3^. There is also no specific investigation for diagnosing a hamartoma. The diagnosis can only be confirmed with HPE; hence, without an excision biopsy, the diagnosis of hamartoma could be missed, as described by Jeffrey in his case series^3^. Surgical removal remains the primary treatment to confirm the diagnosis and alleviate any concerns. However, observation of mass is also an option, especially if it is asymptomatic^3,4^. Still, in this case, the patient is keen about removal. Therefore, an excision biopsy was done, resulting in the discovery of a hamartoma. Recurrence may occur due to incomplete excision, but is not seen in this case^3^. This report underscores the rarity of finger hamartomas and the necessity of considering them in differential diagnoses for soft tissue masses in the finger.

In conclusion, this case report highlights the rare occurrence of a little finger hamartoma, contributing to the limited body of literature on such cases. Presentation of a hamartoma is unremarkable and indistinctive to common tumours of the finger, such as ganglion cysts or lipomas, hence confirmation can only be made through HPE. Further documentation and research are essential to enhance understanding of this rare condition.

## References

[1] Ober WB (1978). Selected items from the history of pathology: Eugen Albrecht, MD (1872-1908): hamartoma and choristoma.. Am J Pathol..

[2] Ali SA, Mulita F. Hamartoma. [Updated 2023 Mar 14]..

[3] Jeffery CC (1973). Hamartoma in the hand—A simulator.. Hand..

[4] Tahiri Y, Xu L, Kanevsky J, Luc M (2013). Lipofibromatous hamartoma of the median nerve: a comprehensive review and systematic approach to evaluation, diagnosis, and treatment.. J Hand Surg Am..

[5] Tjarks BJ, Gardner JM, Riddle ND (2019). Hamartomas of skin and soft tissue.. Semin Diagn Pathol..

